# S5-4 Aquatic literacy for a lifelong safe and fun engagement in aquatic activities

**DOI:** 10.1093/eurpub/ckad133.026

**Published:** 2023-09-11

**Authors:** Kristine de Martelaer

**Affiliations:** Vrije Universiteit Brussel, Belgium

## Abstract

**Development:**

Aquatic literacy (AL), emerging from the broader concept of physical literacy and translated to the aquatic environment, comes close to the concept of water competence. Water competence is described as a broad spectrum of competencies required to effectively help to prevent drowning by integrating the physical competencies with the cognitive and affective. Dudley (2019) explains the learning’ in aquatic environments from a physical literacy perspective, describing four dimensions: psychomotor, cognitive, affective, and social aspects. In aquatic literacy special attention is given to positive motivation to engage in aquatic recreation, considering the degree to which individuals have been privileged or restrained for different aquatic activities in a variety of contexts.

The European project “Aquatic literacy for all Children” or ALFAC aims to improve the quality of aquatic education, in order to prepare them to take part in wide range of water sports while at the same time protect children from the dangers looking for balanced ‘challenges’.

**Implementation:**

Developing children's aquatic skills, motivation, enjoyment, engagement, and confidence will be studied in seven European countries. The target group consists of elementary school children.

An international database, enabling situating the level of children on the basis of reference values is a valuable tool for pedagogues, researchers and institutional actors whose aim is to improve swimming education systems.

**Evaluation & Dissemination:**

The objective of building a standardized set of tests and a common evaluation will allow to build the foundations of a transnational work that will have repercussions on the national plans of aquatic recreation in general and school swimming in particular. The three elements of the Personal Assets Framework are inspiring for the educational approach in aquatics: (a) personal engagement in activities, (b) quality social dynamics, and (c) appropriate settings.

**Conclusion:**

With ALFAC a pragmatic but evidence based international AL measurement tool will be created enabling to identify the most relevant areas of progress in aquatic education. It will offer educational resources proposing tasks, technical content, and educational support to help individual children and groups in their progress of AL.

